# Enhancing glass ionomer cement features by using the calcium phosphate nanocomposite

**DOI:** 10.1590/0103-6440202204887

**Published:** 2022-06-24

**Authors:** Ana Caroline Alves Duarte, Rodrigo David Fernandes Cunha Pereira, Sandhra Maria de Carvalho, Adriana Gonçalves da Silva, Cíntia Tereza Pimenta de Araújo, Rodrigo Galo, Vitor César Dumont

**Affiliations:** 1 Department of Pediatric Clinics, Federal University of the Vales do Jequitinhonha e Mucuri - UFVJM, Diamantina-MG, Brasil.; 2 Federal University of the Vales do Jequitinhonha e Mucuri- UFVJM, Diamantina-MG, Brasil.; 3 School of Dentisry, Federal University of Minas Gerais-UFMG, Belo Horizonte-MG, Brasil; 4 Faculty of Dentistry of Sete Lagoas-FACSETE, Sete Lagoas-MG, Brasil; 5 Department of Dental Materials, Federal University of the Vales do Jequitinhonha e Mucuri - UFVJM, Diamantina-MG,Brasil.; 6 Department of Prosthodontics and Dental Materials, School of Dentistry of Ribeirão Preto, University of São Paulo Ribeirão Preto-SP, Brasil.

**Keywords:** Composites, Chemical Synthesis, Electron Microscopy

## Abstract

This study showed the synthesis of Glass ionomer cements (GIC) modified with calcium phosphate nanoparticles (nCaP). The nCaP/GIC were submitted to mechanical compression and diametral tensile tests. The biocomposite were characterized by scanning electron microscopy (SEM), energy-dispersive X-ray spectroscopy (EDX), X-ray diffraction (XRD) and Fourier-transform infrared spectroscopy (FTIR). Cytotoxicity and cell viability tests were performed on the human bone marrow mesenchymal stem cells using a 3-(4,5-dimethylthiazol-2yl)2,5-diphenyl- tetrazolium-bromide assay and LIVE/DEAD assays. Statistically significant differences were observed for mechanical properties (Kruskal-Wallis, p<0.001), nCaP/GIC showed higher resistance to compression and diametral traction. The SEM analyses revealed a uniform distribution nCaP in the ionomer matrix. The EDX and XRD results indicated that hydroxyapatite and calcium β-triphosphate phases. The FTIR spectra revealed the asymmetric band of ν3PO43- between 1100-1030cm-1 and the vibration band associated with ν1PO43- in 963cm-1 associated with nCaP. The nCaP/GIC presented response to adequate cell viability and non-cytotoxic behavior. Therefore, the new nCaP/GIC composite showed great mechanical properties, non-cytotoxic behavior, and adequate response to cell viability with promising dental applications.

## Resumo

Este estudo apresenta a síntese de cimentos de ionômero de vidro (GIC) modificados com nanopartículas de fosfato de cálcio (nCaP). Os nCaP / GIC foram submetidos a ensaios mecânicos de compressão e tração diametral. Os biocompósitos foram caracterizados por microscopia eletrônica de varredura (MEV), espectroscopia de energia dispersiva de raios-X (EDX), difração de raios-X (XRD) e espectroscopia de infravermelho com transformada de Fourier (FTIR). Os testes de citotoxicidade e viabilidade celular foram realizados em células-tronco mesenquimais da medula óssea humana usando um ensaio de 3- (4,5-dimetiltiazol-2-il) 2,5-difeniltetrazólio-brometo e ensaios LIVE / DEAD. Diferenças estatisticamente significativas foram observadas para as propriedades mecânicas (Kruskal-Wallis, p <0,001), nCaP / GIC apresentou maior resistência à compressão e tração diametral. As análises de SEM revelaram uma distribuição uniforme de nCaP na matriz do ionômero. Os resultados de EDX e DRX indicaram fases de hidroxiapatita e β-trifosfato de cálcio. Os espectros de FTIR revelaram a banda assimétrica de ν3PO_4_^3-^ entre 1100-1030cm^-1^ e a banda de vibração associada a ν1PO_4_^3-^ em 963cm^-1^ associada a nCaP. O nCaP / GIC apresentou resposta adequada à viabilidade celular e comportamento não citotóxico. Portanto, o novo compósito nCaP / GIC apresentou ótimas propriedades mecânicas, comportamento não citotóxico e resposta adequada à viabilidade celular com promissoras aplicações odontológicas.

## Introduction

Glass ionomer cements (GIC) have become a prominent material in dentistry being widely used for aggregating satisfactory physical and biological properties. Adhesion to tooth structure characterized by the chemical interaction of carboxyl groups of polyacids to calcium ions of dental tissues, low contraction and expansion during prey reaction and thermal expansion coefficient similar to that of tooth structure minimize microleakage at the tooth/restoration interface ^1^ ^,^ ^2^ ^,^ ^3^. Its anti-cytogenetic nature associated with biocompatibility and fluoride release act on the remineralization of dental tissues and on the control of caries recurrence, discarding the need for total removal of infected and softened dentin to control the progression of dental caries ^2^ ^,^ ^3^.

The use of GIC as a direct restorative material presents some limitations associated with its low mechanical resistance (abrasion and flexural), friability, high modulus of elasticity and deterioration in acidic pH, being therefore fragile and prone to fracture ^2^ ^,^ ^3^ ^,^ ^4^. Modifications of the glass ionomer cements with metals, polymers and ceramics in different metric scales have been proposed with the aim of improving the mechanical and biological properties ^3^ ^,^ ^5^ ^,^ ^6^.

Calcium phosphate-based (CaP) biomaterials are of special interest because they mimic the main inorganic component of bone, because they present bioactivity and establish an intimate and functional relationship with adjacent bone tissue WANG et al.,^7^.In this context, the use of calcium phosphate (CaP) biomaterials is of particular interest in improving mechanical properties, incorporating topographic features at the nanoscale that mimic the natural tooth nanostructure and establishing an intimate and functional relationship with adjacent tissue ^8^ ^,^ ^9^. The amount of calcium phosphate incorporated requires compatibility between the nanoparticles and the polymer matrix, significantly influencing the wettability and viscosity of the composite ^10^.

Numerous studies with calcium phosphate particles added to composites demonstrated improvements in mechanical properties ^1^ ^,^ ^3^ ^,^ ^4^ ^,^ ^5^ ^,^ ^6^ ^,^ ^10^. However, no study has been reported in the literature involving the hydroxyapatite and β-TCP phases of calcium phosphate in the modification of glass ionomer cement for the improvement of mechanical properties and decrease of cytotoxicity.

This study is reported to biocomposites synthesis based on arrays of glass ionomer cement (GIC) modified with calcium phosphate nanoparticles (nCaP). The biocomposites were characterized by scanning electron microscopy (SEM), energy-dispersive X-ray spectroscopy (EDX), X-ray diffraction (XRD) and Fourier transform infrared spectroscopy (FTIR). Cytotoxicity and cell viability tests were performed on the human bone marrow mesenchymal stem cells (HBMSC) using a 3-(4,5-dimethylthiazol-2yl) 2,5-diphenyl tetrazolium bromide (MTT) assay and LIVE/DEAD assays. The hypothesis is that the combination of GIC with nCaP nanoparticles would produce a compound with improved mechanical properties, reduced cytotoxicity, and good viability response.

## Materials and Methods

### Materials

All the reagents and precursors, phosphoric acid (Sigma-Aldrich, USA, 85%, H_3_PO_4_), calcium hydroxide (Sigma-Aldrich, USA, ≥96%, Ca(OH)_2_) and ammonium hydroxide (Synth, Brazil, 30%, NH_4_OH) were used as received. Ionomer glass cement (GIC) (FGM, Brazil, Maxxion R) was modified. Deionized water (Millipore Simplicity™) with a resistivity of 18MΩ cm was used in the preparation of all solutions. Potassium bromide (Sigma-Aldrich, USA, ≥99%, KBr), suitable for spectroscopy, was used to prepare the FTIR pellets.

### Synthesis of calcium phosphate nanoparticles (nCaP)

**Figure 1:**
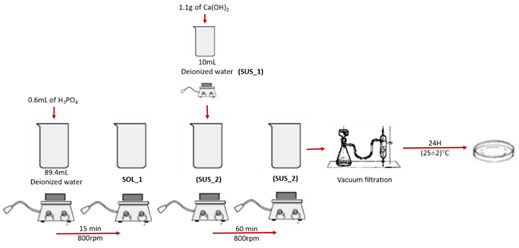
Synthesis of calcium phosphate nanoparticles (nCaP)

The nCaP particles were synthesized by aqueous precipitation route, at (25±2)°C (Figure 1) ^10^. The precursors were prepared as follows: 0.6ml of H_3_PO_4_ was slowly added to 89.4ml of deionized water under magnetic stirring for 15min. This phosphate precursor solution was referred to as "SOL_1".

Approximately 1.1g of Ca (OH)_2_ powder was added to 10mL of deionized water and under vigorous stirring for 15min. This calcium suspension was referred to as "SUS_1". Then "SUS_1" was added slowly to "SOL_1" for the synthesis reaction and this mixture ("SUS_2") was magnetically stirred for 1h.

Subsequently, SUS_2 was allowed to stand for 24h at (25±2)°C. The supernatant was decanted from the solid material. The precipitate was vacuum filtered using custom filter paper on a Büchner funnel, 3 washes were performed with deionized water and filtered again. The material retained was subjected to drying at a temperature of (25±2)°C for 96h. The chemical reaction of the formation of calcium phosphate is represented in the equation 1:

(Eq. 1)

$$\;6H_{3}{PO}_{4}(aq)\;+\;10\;{Ca(OH)}_{2}(sus)\;\to \;{Ca}_{10}({PO}_{4})O_{6}{\left(OH\right)}_{2}(s)\;+\;18\;H_{2}O$$ (1)

### Biocomposite synthesis (nCaP/GIC)

The nCaP/GIC was obtained by the addition of 1.1g of nCaP which were weighed and added to 10g powder of the GIC. The agglutination of the material followed the standards required by the manufacturer in room temperature (25±2)°C.

### Mechanical tests

Test specimens (cps) (n=20) of GIC and nCaP/GIC were made in a teflon matrix with 4mm in diameter and 8mm in length, resting on a glass plate. The cement was inserted into the matrix under pressure through a specific syringe (Centrix, DFL Ind., São Paulo, SP, Brazil) to minimize the formation of bubbles in the cement body. After complete filling of the matrix, a polyester strip was pressed on the surface of the cement under a weight of 500g until reaching its setting time in order to obtain adequate flow and surface smoothness of the material. After 24h of storage in distilled water, at (37±1)°C, cps (n=10) of G1-GIC and G2- nCaP/GIC were subjected to the compressive strength test in a universal test machine EZ Test (Shimadzu, Japan) with a load cell of 200kgf at a speed of 1mm/min, with its long axis in the vertical position, until its fracture. For the diametral tensile strength test, cps (n=10) was submitted to the same load cell, but with a velocity of 0.5mm/min and with its long axis in the horizontal position.

The results were submitted to the normality test (Shapiro-Wilk, p≥0,05) then a parametric statistical test (ANOVA) was applied to verify differences between the groups using the Statistical Package for Social Sciences (SPSS for Windows, version 17.0, SPSS Inc., USA). Statistical analysis of the data was performed with a level of significance of 95%.

### Characterizations of the biocomposite (nCAP/GIC) and precursors

*Scanning electron microscopy (SEM) and energy dispersion X-ray spectroscopy analysis (EDX).*

The morphologies glass ionomer cement (GIC) modified with calcium phosphate nanoparticle (nCaP) were evaluated using a scanning electron microscope (SEM, FEI-INSPECTTM S50) coupled with energy dispersion X-ray spectroscopy (EDX, EDAX GENESIS). Before examination, the samples were coated with a thin carbon film via sputtering using a low deposition rate, cooling the substrate, and ensuring the maximum distance between the target and the sample to avoid sample damage. Images of secondary electrons (SE) were obtained using an accelerating voltage of 15kV.

The nCaP particles sizes and size distribution data were obtained based on the SEM images by measuring at least 100 randomly selected nanoparticles using an image processing program (Image J, public domain software, version 1.44, National Institutes of Health).

###  X-ray diffraction (XRD) 

The crystallinity of the phases presents in the biocomposites (nCaP/GIC) was assessed based on the X-ray diffraction (XRD) patterns recorded using a PANalytical X'Pert diffractometer (Cu-Kα radiation with λ=1.5406Å). Measurements were performed in the 2θ range of 15° to 75° with steps of 0.06°.

###  Fourier-transformed infrared spectroscopy (FTIR) 

 Fourier transform infrared (FTIR) was performed in the range of 650 to 4000cm-1 (Fischer Thermo Nicolet 6700) using the transmission mode. The nCaP and nCaP/GIC were placed in a sample holder and scanned immediately (16 scans) with a resolution of 2cm^-1^ background subtraction. The samples were mixed in a proportion of 1% (% by weight) into dry KBr powder at (110±5)°C for 2h. The FTIR spectra of films and biocomposites were obtained using attenuated total reflectance (ATR, 4000-675cm-1 using 32 scans and a resolution of 4cm^-1^) with background subtraction.

### Cytotoxicity assay - Culture of cells

###  Human bone marrow mesenchymal stem cells 

Culture of human embryonic kidney lineage cells (T HEK 293 cells) and human bone marrow mesenchymal stem cells (HBMS). The cells were cultured in Dulbecco’s modified eagle medium (DMEM) with 10% fetal bovine serum (FBS) penicillin G sodium (10units.mL-1), streptomycin sulfate (10mg.mL-1) and amphotericin-b (0.025mg.mL-1) all from Gibco BRL (NY, USA) in a humidified atmosphere of 5% CO_2_ at (37±1) °C. The cells were used for experiments on passage 5. The cells used for the experiments were from passages 23, 12 and 4 for SAOS, HEK 293 and HBMS, respectively.

Toxicity assay by resazurin and MTT: All biological tests were conducted according to ISO standards 10993-5:1999 (Biological evaluation of medical devices)

###  (3-(4,5-dimethylthiazol-2yl) 2,5-diphenyl tetrazolium bromide) MTT assay 

 HBMSC cells were plated (3×104 cells/well) in 96-well plates. Cell populations were synchronized in serum-free media for 24h. After this period, the medium was aspirated and replaced with medium containing 10% FBS. Samples of GIC and nCaP/GIC (5mg.mL^-1^) were added to individual wells. Controls were used with the cells and DMEM with 10% FBS, the positive control Triton x-100 (1% v/v in phosphate buffered saline, PBS, Gibco BRL, NY, USA) and, as a negative control, chips of sterile polypropylene Eppendorf tubes (1mg.mL-1, Eppendorf, Hamburg, Germany). After 72h, the medium was aspirated and replaced with 60µL of culture medium with serum in each well. Next, 50µL of MTT medium (5mg.mL-1) (Sigma-Aldrich, 131MO, USA) was added to each well and was incubated for 4h in an oven at (37±1)°C and 5% CO_2_. Subsequently, 40µL of the SDS solution/4% HCL was placed in each well and incubated for 16h in an oven at (37±1) °C and 5% CO_2_. Then, 100µL was removed from each well and transferred to a 96-well plate to quantify the absorbance (Abs) using Varioskan Reader (Thermo Scientific) with a 595-nm filter. The values obtained were expressed as percentage of viable cells according to the following formula: Cell viability (%) = (absorbance samples and cells x 100) / absorbance (control). Assume the values of controls (wells with 137cells, and no samples) as 100% cell viability.

Prism software (GraphPad Software, San Diego, CA, USA) was used for data analysis. Statistical significance was tested using One-way ANOVA followed by Bonferroni test. A p value < 0.05 was considered statistically significant (n=3).

###  Live / dead assay 

HBMSC cells were plated (3×104 cells/well) in 96-well plates. Cell populations were synchronized in serum-free media for 24h. After this period, the medium was aspirated and replaced with medium containing 14510% FBS. Samples of GIC and nCaP/GIC (5mg.mL^-1^) were added to individual wells. After 72h, all media was aspirated, washed with PBS for two times with 10mL of phosphate buffered saline (PBS) from (Gibco 147BRL, NY, USA). The HBMSC cells were treated for 30min with the kit LIVE / DEAD Viability / cytotoxicity from (Life Technologies of Brazil Ltda, São Paulo) according to manufacturer's specifications.

Images were obtained with an inverted optical microscope (Nikon, Japan), the fluorescence emissions should be acquired separately as well, calcian at 530 ± 12.5nm, and EthD^-1^ at 645 ± 20nm.

## Results

The results for the mechanical test were a statistically significant difference between GIC and nCaP/GIC for the compressive strength test and the diametral tensile strength test (table 1).

**Table 1:** Mean values (MPa) of the compressive strength (CS) and diametral tensile strength (DTS).

Specimens	CS	P*	DTS	P*
GIC	21,8±6	p=0,002	24,4±7	p<0,001
nCaP/GIC	39,2±4		47,1±4	

**Figure 2:**
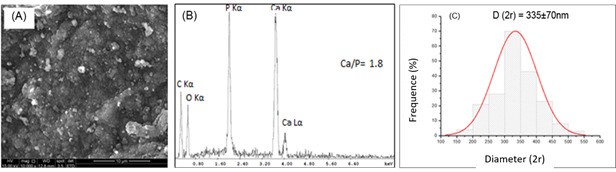
Morphological analysis of nCaP ((A) SEM **image**). Chemical analysis: EDS spectra (B) and histogram of the mean size of nCaP (C).

Morphological evaluations of nCaP show considerable heterogeneity in the form of the synthesized particles (Figure 2(a)), indicating morphological aspects for the formation of nanoparticles with sizes ranging from 50-100nm. The characteristic EDX spectra are shown in Figure 2(b) showing peaks associated with Ca and P elements, and a Ca/P ratio equal to 1.8, suggesting the precipitation of the hydroxyapatite phase. The synthesis process allowed the formation of particles in the gauge scale (Figure 2(c)).

**Figure 3:**
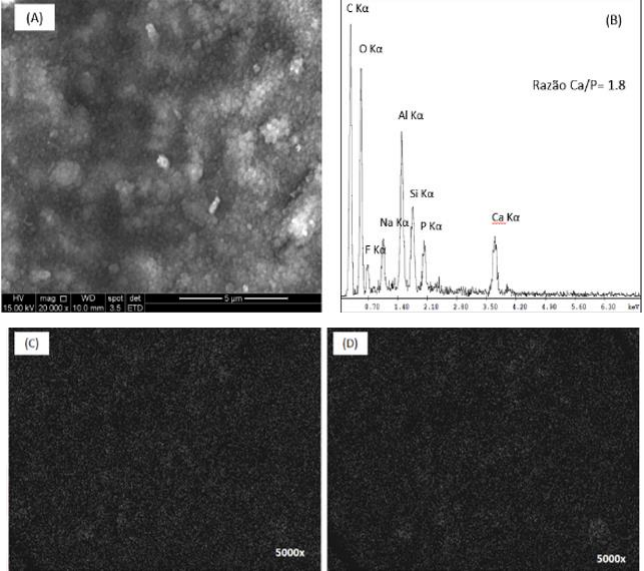
Morphological analysis of nCaP/GIC ((A) SEM image). Chemical analysis: EDS spectra (B) and **mapping** of Ca Kα (C) and P Kα (D) elements.

The modification of the GIC allowed the synthesis of homogeneous biocomposites with greater surface roughness (Figure 3(a)). The EDX spectra showed peaks of Ca and P elements attributed to nCaP as shown in Figure 3(b). In addition, the Ca-Kα mapping analyzes revealed that the particles of nCaP are uniformly dispersed in the composite matrix without detecting any segregation (Figures 3(c) and 3(d)).

**Figure 4:**
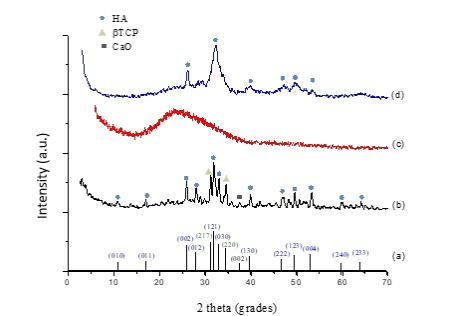
XRD spectra of **the** reference CaP (ICDD-96-900-3549) (A), nCaP (B), GIC (C) and nCaP/GIC (D).

The standard calcium phosphate (nCaP) XRD, GIC and nCaP/GIC are shown in figure 4. The figure 4(a) showed characteristic peaks of calcium phosphate particles (International Centre for Diffraction Data, JCPDS 86-1203). The XRD spectra of nCaP (Figure 4(b)) showed major peaks characteristic of HA in 2 theta equal to 31,7º ( 2 1 1) 32,8º (3 0 0) 32,2º (1 1 2), and 25,9º (0 0 2), and other smaller peaks with intensities associated β - tricalcium phosphate (β -TCP) phase (28.0◦, 31.2◦, and 34.5º). The XRD spectra of nCaP/GIC (Figure 4(c)) showed of the halo characteristic of polymers with amorphous appearance. X-ray diffraction also certifies that the GIC overlaps the characteristic peaks of β-TCP, evidencing only the peaks associated with hydroxyapatite (Figure 4(d)).

**Figure 5:**
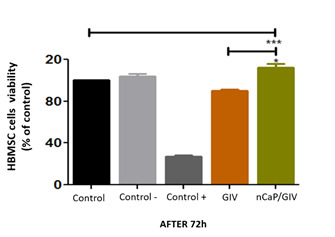
MTT assay after 24h incubation direct contact with mesenchymal stem cells from bone marrow. (Control; Control -; Control +; GIV; nCaP/GIV).

The figure 5show the spectra of nCaP/GIC. The FTIR results shown the asymmetric band of ν3 PO^3-^ between 1100-1030cm^-1^ and the vibration band associated with ν1 PO^3-^ in 963cm^-1^ associated with the phases of calcium phosphate.

**Figure 6:**
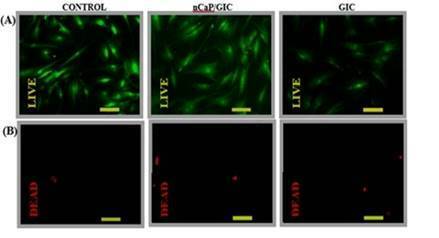
Live/Dead assay with HBM cells after 72h of direct contact. In the control, in the GIC and nCaP/GIC samples (bar = 100 μm, 200x), live cells ((A), green) and dead cells ((B), red).

 When analyzing the behavior of HBMSC cells in contact with the GIC sample showed no significant difference in viability when compared to the control group. However, when analyzing the cells in contact with the nCaP/GIC sample there was a significant increase in viability of 12.00 ± 6.50% when compared to the control group. Moreover, when we compare the behavior of HBMSC cells in contact with the two biomaterials observed significant difference of 22.00% in nCaP/GIC sample compared to GIV sample (Figure 6).

## Discussion

Efforts are taken to improve the performance of dental cavity restorations (12). The process of the tooth structure demineralization refers to the dissolution of calcium and phosphate ions by saliva, while remineralization refers to the mineral precipitation ^13^. Thus, an important approach to inhibit demineralization and to promote remineralization was the development of calcium phosphate-based materials ^14^. These compounds release Ca and PO_4_ ions to supersaturating levels in relation to the tooth mineral, and they have been shown to protect teeth from demineralization, or even to regenerate lost tooth mineral in vitro ^12^. 

Compressive strength is an important characteristic in the evaluation of dental materials, as it indicates the materials resistance to forces those teeth are repeatedly subjected to during mastication ^15^. A previous study had shown that the addition of hydroxyapatite nanoparticles to the glass ionomer powder was promising, increasing compressive strength and the diametrical traction. This corroborates the present study in which nCaP was added to the glass ionomer powder, and the tests results showed that both glass powders had higher strength values ^16^. However, other researchers Dionysopoulos et al., ^17^ observed that a slight reduction in the compressive strength of GICs tested, when the CaCl_2_ were used, but this reduction was not statistically significant indicating that the tested treatment was not detrimental for the GICs.

The results of the nCaP/GIC in the mechanical assays may be associated with a strong influence of the increase of the calcium concentration in the prey steps of the material. In the initial stage during the agglutination of the powder and liquid, the hydrogen promotes the displacement of calcium and aluminum ions that react with the fluoride forming calcium and aluminum fluorides. As the pH of the system decreases the dissociation of these fluorides occurs which react with the copolymers forming more stable complexes ^18^. It is suggested that the increase in calcium concentration by the incorporation of nCaP displaces the chemical reaction a favor of the formation of these more stable compounds ^19^.

Another step that can be influenced by the incorporation of nCaP is the formation phase of the polyacid matrix. At this stage, the release of calcium occurs with greater velocity due to its cationic character interacting with the aqueous chains of polyacids forming crosslinks, forming the gel matrix allowing hardening of the material ^20^. Thus, the high concentration of calcium can accelerate the prey of the material minimizing the influence of the medium, syneresis and imbibition, predisposing the nCaP/GIC to present better results in the mechanical tests ^21^.

Similar to all other restorative materials, modifying the size and shape of particles added to a GIC can influence its mechanical properties ^22^. Generally, a smaller particle size and higher filler improves the compressive strength and hardness of GICs, while larger particles can lead to greater wear resistance ^23^. Modified glass ionomers have greater flexural strength, tensile strength and solubility resistance compared to conventional GICs, which may be due to the chemical bond between the glass particles and the resin phase ^24^ ^,^ ^25^.

The peaks showed by the FTIR of the nCaP/GIC nanoparticles were in agreement with previous studies in which the OH peak was not present and the broad peak in the 1100 cm^-1^ region was observed ^26^. Regarding the nanoparticles, the 963cm^-1^ bands correspond to the vibration modes of the tetrahedral phosphate (PO_4_^3−^). The FTIR spectra should include characteristic bands from both GIC and nCaP. However, because of the higher proportion of the glass phase weight compared to the modified GIC, the absorption bands related to the nCaP are covered by the GIC bands and are not identified in the spectra, confirming the presence of amorphous structure ^27^.

The incorporation of 5.0% by weight of HA nanoparticles in GIC, after acid attack on ceramic particles, showed more available Ca^2+^ ion for cement curing through bridge formation, which would reinforce the GIC matrix ^28^. Furthermore, according to Moshaverinia et al. ^27^ GICs become stronger as they mature after one and seven days of storage in distilled water at 37° C.

The XRD pattern of the GIC and nCaP/GIC showed a wide diffraction in the 2θ = 25.9-32.8° range, which is characteristic of the amorphous glass phase ^29^. The nCaP nanoparticles also showed a small characteristic diffraction located at 2θ = 28-34.5° ^28^. Because of the nCaP nanoparticles incorporation in the glass ionomer powder, the peak center changes to higher degrees compared to the conventional glass ionomer, which is related to the presence of nCaP in the glass.

In the current study the viability of HBMSC cells in direct contact with GIC and nCaP/GIC samples were analyzed by MTT assay. This test is specifically used to evaluate mitochondrial function and cell viability. It can be seen that the HBMSC cells in contact with both samples showed similar patterns of fluorescence when compared to the untreated control group, ie, high green fluorescence (viable cells) and little or no red fluorescence (dead cells). The similarity of the fluorescence is more evident in cells in contact with the nCaP/GIC sample.

Studies indicate that the GIC cytotoxicity is dependent of the dose or residual products, such as HEMA and, as far as is known, few studies have reported the cytotoxicity and biocompatibility of the nCaP incorporated to GIC ^30^. The cell viability demonstrated by nCaP was comparable with the control group and it showed a significant increase over the control group. Thus, the nCaP cell viability seems to exhibit favorable and similar results to the GIC, demonstrating noticeable results when the nCaP is incorporated. A previous study in which nano-HA cytotoxicity was tested before GIC incorporation, showed similar results, reporting moderate to low cytotoxicity and no genotoxicity at its highest concentration (100 mg/ml) ^31^.

This study demonstrates for the first time that the incorporation of nanoparticles of calcium phosphate, hydroxyapatite and BTCP in nanometric scale affect the GIC prey phases. The results showed that nCaP was evenly distributed in the ionomer matrix and provided improvements in the mechanical properties of the material. Regarding cytocompatibility in vitro, no toxicity was observed for any of the groups tested. The nCaP/GIC biocomposites are more promising for potential application in dentistry.
